# Open Data for Differential Network Analysis in Glioma

**DOI:** 10.3390/ijms21020547

**Published:** 2020-01-15

**Authors:** Claire Jean-Quartier, Fleur Jeanquartier, Andreas Holzinger

**Affiliations:** Holzinger Group HCI-KDD, Institute for Medical Informatics, Statistics and Documentation, Medical University Graz, Auenbruggerplatz 2/V, 8036 Graz, Austria; f.jeanquartier@hci-kdd.org (F.J.); a.holzinger@hci-kdd.org (A.H.)

**Keywords:** open data, cancer research, differential network analysis, differential gene expression, protein-protein interaction, graph-based analysis, glioma, glioblastoma multiforme, astrocytoma, biological data integration

## Abstract

The complexity of cancer diseases demands bioinformatic techniques and translational research based on big data and personalized medicine. Open data enables researchers to accelerate cancer studies, save resources and foster collaboration. Several tools and programming approaches are available for analyzing data, including annotation, clustering, comparison and extrapolation, merging, enrichment, functional association and statistics. We exploit openly available data via cancer gene expression analysis, we apply refinement as well as enrichment analysis via gene ontology and conclude with graph-based visualization of involved protein interaction networks as a basis for signaling. The different databases allowed for the construction of huge networks or specified ones consisting of high-confidence interactions only. Several genes associated to glioma were isolated via a network analysis from top hub nodes as well as from an outlier analysis. The latter approach highlights a mitogen-activated protein kinase next to a member of histondeacetylases and a protein phosphatase as genes uncommonly associated with glioma. Cluster analysis from top hub nodes lists several identified glioma-associated gene products to function within protein complexes, including epidermal growth factors as well as cell cycle proteins or RAS proto-oncogenes. By using selected exemplary tools and open-access resources for cancer research and differential network analysis, we highlight disturbed signaling components in brain cancer subtypes of glioma.

## 1. Introduction

The understanding of complex diseases such as cancer requires insight into high dimensional data, underlying domain knowledge and multiple networks that provide relevant relationships among biological entities [1]. We thereby introduce the topics of open data, biological data in the context of gene expression and protein interactions, the approach of graph-based analysis, as well as the biological background for signaling in exemplary cancer diseases of glioma.

### 1.1. Web Resources and Open Data

Cancer databases include various data types ranging from biological and pharmacological data, including information on genome, proteome as well as metabolome, and clinical information on experiments, incidence, mortality, prevalence and survival. Common issues of data retrieval are access restrictions or poor annotation due to sharing reluctance and fear about lack of control over its usage and personal credit [2]. Still, the long history of producing data has been revolutionized and shaped its form of being laborious, costly and fragmentary to so-called big data generated by high throughput techniques and large multi-site collaboration projects [3]. This open sharing of data requires structures to secure common guidelines to data formats, standards, meta-data, intellectual property rights, licensing and sharing protocols. There are a wide range of datasets available open for data mining, predictive modeling or other purposes [4]. Obtainable data enable many hypotheses to be tested in silico, saving time and money, and maximizing efficiency [5]. Various data resources can be of interest including biomolecular repository hubs, additionally, offering not exclusively upload and analysis options or links to publications. In order to avoid discrepancies, one has to rely on globally normalized and quality controlled public experiments [6]. Biomolecular data types include cancer-related whole genome and large-scale genomic sequencing data, copy number alterations, DNA-methylation, different types of mutations, microarray data, microRNAs, RNA sequencing data, protein–protein interaction (PPI) probing, protein mass spectrometry, drug-target relationships, further biological and pharmacological data, as well as cancer incidence, mortality rates, prevalence and survival rates.

In this paper, we focus on gene expression and PPI data.

### 1.2. Gene-Expression Data

The unique pattern of gene expression for a given cell or tissue is referred to its molecular signature and defines a particular class of tumor.

Each subtype holds distinct biological properties affecting clinical matters, such as metastasis likelihood and patient survival prognosis as well as tailored therapy strategies. Differential gene-expression data can be used for deriving marker genes indicating multiple types of cancers or specific to individual cancers [7]. In reference to the latter group, signaling pathways have been pointed out to be consistently and highly enriched across different types of cancers, such as Wnt, p53 and integrin signaling pathways, as well as others like phospho-APC/C-mediated degradation of cyclin A and inflammation determined by chemokine and cytokine signaling pathways. The co-occurrence of alterations in these pathways may differ between individual tumors and tumor types [8].

Differential gene expression data can be aligned to differences between cell-lines, wild-type and mutated or knock-outs, between disease and control samples or simply different disease subtypes. Gene expression is mostly assessed by transcriptomic methods [9]. Whole transcriptome analysis allows thousands of genes to be studied at once. The sheer abundance of generated data by high performance methods makes it difficult to encompass all contents. In detail, problems are raised for pre- and post-processing steps such as gene mapping, data cleaning, storage, retrieval and analysis infrastructure. The analysis requires the steps of image analysis of the microarray, normalization methods, data analysis through estimation, testing, clustering, discrimination and its biological verification and interpretation. There are several challenges, including sequence reannotation, alternative splicing, or simply numerical data interpretation. It has to be kept in mind that using data from microarray experiments is based on various assumptions. These include the direct relation of protein to mRNA content, equal mRNA capturing proficiency by the applied method, no impact from perturbations and foremost by no means on housekeeping genes. The different experimental settings and methods generate heterogeneous data sets that can be hardly merged and is rarely annotated. These aspects ask for a unifying framework, including standardizing methods. Official gene expression repositories are, to name a few, Expression Atlas [10], ArrayExpress [11], Gene Expression Omnibus [12], dbGAP [13], the European Genome-phenome Archive [14], IntAct [15], Japanese Genotype-phenotype Archive [16], Biological General Repository for Interaction Datasets (BioGRID) [17], NCBI PubChem BioAssay [18], cBioPortal [19], and many others, and involve the implementation of MIAME standards, the so-called minimum information about a microarray experiment. Since EMBL-EBI’s Expression Atlas [10,20] provides gene expression from many experiments that are highly curated and normalized, we use data from this database to base our study on.

Identifying patterns of gene expression supports characterizing subtypes of cancer towards a more personal treatment approach, such as in the case of glioblastoma [21]. Discrepancies when comparing cancer cells with normal cells arise from the utilization of heterogeneous cell mixtures of the organ in question instead of single cell samples of cancer cells and their nearest benign neighbor cells [22]. Recently, a framework was published for partitioning the variation in gene expression due to a variety of molecular variables, including somatic mutations, transcription factors, microRNAs, copy number alternations, methylation and germ-line genetic variation [23]. Meta-analysis allows for expression data from various public repositories to be merged in order to make group comparisons that have not been considered before [24]. Recently, a web-based application was published which can be used to download, collect and manage gene expression data from public databases [25]. Integrating gene expression data and PPI networks has been presented for the prediction of essential proteins [26].

### 1.3. PPI Networks and Graph Analysis

Protein interactions determine cellular communication based on signal transduction cascades. These resemble molecular circuits consisting of receptor proteins, kinases, primary and secondary messengers which modulate the gene transcription or the activity of other proteins. Databases provide disease-specific as well as general PPI information [27,28,29,30,31]. PPI data can be based on wet experimentation as well as prediction [32,33]. Integrative tools for visualization of PPI networks thereby facilitate exploration and analysis tasks.

PPI networks are commonly modeled via graphs whose nodes represent proteins and whose undirected edges connect pairs of interacting proteins [34]. These graphs easily comprise large quantities of elements in a four to five-digit range or even more. Analysis thereof often requires automated methods for subnetwork identification with topological or functional characteristics [35]. Visual representation of interaction networks has the objective to give a review on data and to reveal otherwise hidden patterns by augmenting human cognition in order to make sense of often large quantities of abstract information [36]. Graphs can be visualized by modern approaches mapping abstract data by visual transformation and subsequent rendering, such as the prefuse force-directed layout [37].

There are several graph-level features that suit the comparison task according to the existing literature on graph comparison [38,39,40]. Network analysis comprises several computations based on the number of network elements such as nodes and edges, the network diameter, path length, clustering coefficients, degrees and shared neighbors as well as computational construction of differential subnetworks. Thereby, common graph-based algorithms have been developed, such as Molecular Complex Detection (MCODE) [41] or Clustering with Overlapping Neighborhood Expansion (ClusterOne) [42]. Graph-based approaches to cellular network analysis have been comprehensively reviewed before [39,43,44]. Finding clustering entities as separated part of a network also involves outliers that are different from the remaining dataset or overlapping subnetworks with hub nodes belonging to multiple entities. Protein complexes could be identified as being part of dense regions containing many connections in PPI networks, but multifunctional proteins could be part of several clusters [42]. Biological network analysis requires both the topological information and the biological background which is commonly represented by Gene Ontology (GO) terms comprising cellular components, biological or molecular functions.

### 1.4. Signaling Background

Cancer is based on oncogenic mutations resulting in aberrant signaling in tumor cells and effecting increased mitogenesis, prevention of apoptosis, as well as increased cell motility and invasion [45]. Cancerous growth occurs upon an unbalance within genes that control cell proliferation [46]. Cell proliferation is defined by cell cycling behavior and activity, which is known as the growth fraction whereas the term cell growth describes the increase in mass and cell proliferation relates an increase in cell number [47]. We concentrate our study further on genes associated to proliferation. As exemplary class of tumors, we chose to focus on glioma. Glioma comprise different subtypes of glial tumors including astrocytoma and glioblastoma multiforme (GBM). Thereby, the latter group is the most aggressive type and to date, non-curable [48,49]. Astrocytoma derive from star-shaped glia cells that are called astrocytes [50]. Glioma cells firstly experience disrupted pathways of cell cycle control, including mutations in p16, CDK-4, cyclin D1 and RB1 [51]. Pro-apoptotic signals are hindered mostly through tumor protein p53 (TP53) mutations. These tumor cells further exhibit overexpressed growth factors and receptors, such as the transforming growth factor alpha (TGFA) and the epidermal growth factor receptor (EGFR) based on mutations [52]. The vascular endothelial growth factor (VEGF) is further postulated as angiogenic switch and tumor progression [51]. Invasion and migration are modulated by signaling events as the overexpression of extracellular matrix molecules and cell surface receptors. In more detail, glioma can be roughly subdivided into four grades, including low (LGG) and high grade (HGG) types. HGG, grade IV, also called glioblastoma multiforme (GBM) are the most frequent and malignant brain tumor. Thereby, glioblastoma can be distinguished, as primary when developing rapidly without a less malignant precursor, or as secondary, progressing from LGG as astrocytoma [53]. Secondary glioblastoma exhibit mutations in isocitrate dehydrogenase 1 (IDH1) and TP53, uncommon in pediatric malignant glioma [54,55]. IDH1 can be used as biomarker for secondary glioblastomas in addition to several newly identified key biomarker genes, such as PRDX1, based on Support Vector Machine Learning using differentially expressed genes [56]. Our study focuses on GBM in comparison to low-grade astrocytoma and general glioma, that surpasses several diseases within the general group of glioma tumors, including both IDH-wildtype and IDH-mutant glioma.

## 2. Results

We identified NetworkAnalyst [57], Navigator [58], OmicsNet [59], WebGestalt [60] and Cytoscape [61] to freely create and visualize a PPI network for comparison for further analysis of selected genes. Among these, Cytoscape, NetworkAnalyst and OmcisNet are suitable tools for creating protein-coding gene networks based on gene lists. Both NetworkAnalyst and OmicsNet are web-based tools that offer a StringDB interface for calculating PPI, while Cytoscape does not offer a StringDB interface, but, among others, a BioGRID based one. NetworkAnalyst and OmicsNet use the same confidence level of 90% and include only those relations that are already marked with evidence by default. These two tools compute similar results regarding subnetworks, nodes, edges and so called “seed” proteins. Since the calculation is mostly the same, with differences between 2 nodes and edges only, we can assume that the PPI computation is unambiguous. We therefore proceed with only comparing the different results between the graph result of BioGRID and the one of StringDB. Though, StringDB has been shown to be the more comprehensive source [31], the sum of interactions calculated by BioGRID is far more compared to StringDB. This is due to the fact that StringDB-based networks were filtered on a high confidence level of evidence-based information, and BioGRID-based network construction includes all available data without any evidence or confidence filter. In NetworkAnalyst, one can specify the organism human at the beginning. Instead, each constructed BioGrid network included data from all available taxonomies. By filtering on human taxonomy, the networks were thus reduced around 15% in number of nodes.

**Figure 1:**
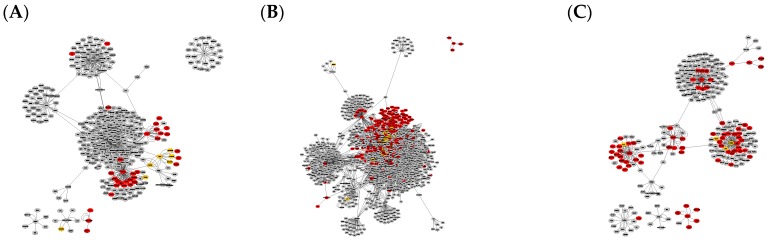
Graph clusters from BioGRID showing unique difference networks between the intersection of all three disease-type constructed networks and each type-specific network with (**A**) general glioma, (**B**) glioblastoma multiforme and (**C**) low-grade astrocytoma. Graphs were rendered with Prefuse Force Directed Layout, clustered by ClusterOne (grey: outlier, yellow: overlap, red: cluster).

**Figure 2:**
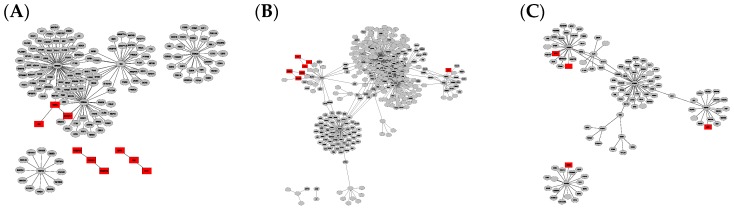
Graph clusters from StringDB showing unique difference networks between the intersection of all three disease-type constructed networks and each type-specific network with (**A**) general glioma, (**B**) glioblastoma multiforme and (**C**) low-grade astrocytoma. Graphs were rendered with Prefuse Force Directed Layout, clustered by ClusterOne (grey: outlier, yellow: overlap, red: cluster).

Constructed PPI graphs from the different network analysis tools using filtered gene expression data from EMBL-EBI Expression Atlas as input are illustrated in Figure S1 for BioGrid-based networks and in Figure S2 for String-DB constructed networks. Networks were then filtered to unique nodes by subtracting the merged intersection of all three disease type-specific networks, illustrated in Figure 1 in case of BioGrid-constructed networks and in Figure 2 based on StringDB. The resulting numbers of nodes and edges as well as calculated parameters from network analysis, including network diameter, clustering coefficient and connected components for each disease- and database-specific network, are summarized in Table 1.

The comparison of graphs in Figure 1 and Figure 2 illustrates that the StringDB-based graphs’ size is far smaller and does not show any potential overlapping protein complexes. This is also true for the comparison of originally constructed networks without subtraction of the intersection equal to all disease subtypes, presented in Figures S1 and S2. All BioGrid-based networks consist of around five times as many elements and interactions compared to those constructed via StringDB, as shown by the number in Table 1. Several subnetworks could be distinguished by cluster analysis via Cytoscape within the larger networks while only a small number of clusters could be observed in graphs based on the latter database.

Merged intersections and unions of the different disease- and database-specific networks as well as their unique differences among each other are summarized in Table 1 and visualized in Figure 1 and Figure 2. Network data exported as cys-files can be found on https://github.com/schokine/Cytoscape-glioma-CF.

### 2.1. Network Analysis of GO Terms and Genes of Interest

The GO-term proliferation has been originally used to filter overexpressed genes within the extensive datasets of each general glioma, glioblastoma multiforme and low-grade astrocytoma. Proliferation is related to malignancy and was thereby chosen as exemplary key term for further analysis. This filtering step was computationally necessary due to the high number of genes which could not properly function as input for the diverse tools without error-free processing. The list of genes was thereby reduced from the magnitude of multiple thousands to less than hundred entries. Filtered gene lists that were used as input for network creation can be found for each of the three glioma subtypes on https://github.com/schokine/Cytoscape-glioma-CF/. Filtered datasets were used to create PPI networks using BioGRID and StringDB, illustrated in Figures S1 and S2.

**Figure 3:**
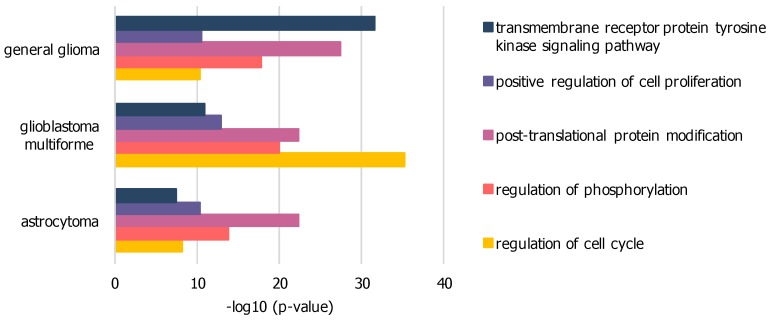
Significant GO-terms within PPI networks of GBM, general glioma and low-grade astrocytoma dissections: based on elevated expression levels of genes associated with GO-term “proliferation”, enriched via BioGRID, difference from merged network intersection; significance expressed as p-value, calculated by BinGO.

The difference networks from unique nodes to each sample dataset, isolated by subtraction from the intersection of all three sample networks, were used for further GO analysis to highlight significant biological functions. Exemplary GO-terms with low p-values within all three sample networks are presented in Figure 3 and Table S1. The general glioma network comprises 219 nodes related to transmembrane receptor protein tyrosine kinase signaling pathway with higher significance compared to other sample networks. The GBM network comprises 446 nodes related to cell cycle regulation at highest significance in comparison to other sample networks. The low-grade astrocytoma network does not further show any biological function unique to its disease subtype in comparison to general glioma or GBM.

**Figure 4:**
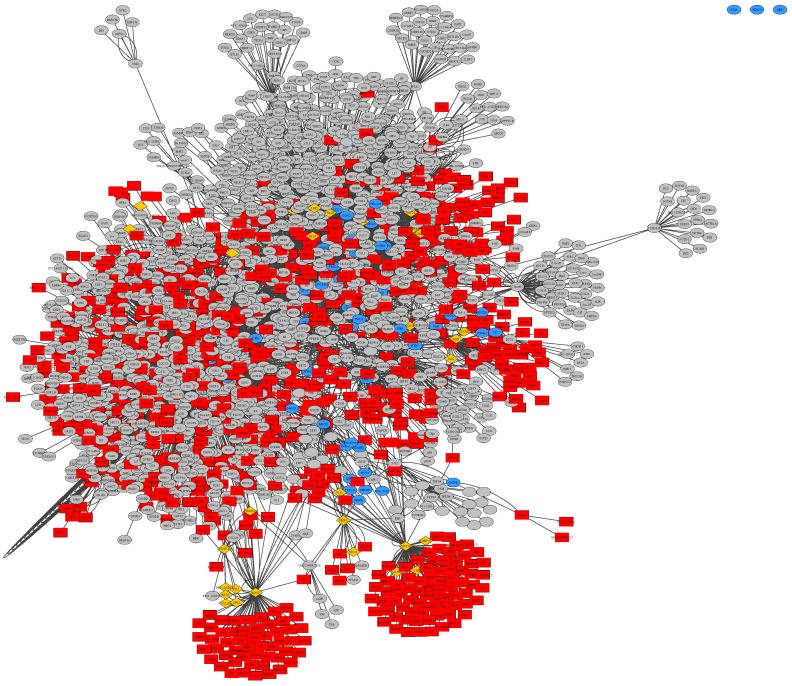
Merged union network of general glioma, glioblastoma multiforme and low-grade astrocytoma samples, constructed from BioGrid in Cytoscape, rendered with Prefuse Force Directed Layout, clustered by ClusterOne (grey: outlier, yellow: overlap, red: cluster, blue: unassigned/default).

Within Figure S1, there are three unconnected nodes common to all three BioGRID based networks, which can be easily recognized within the merged union-network of all three disease subtypes in Figure 4. These nodes represent the protein-coding genes HDAC4 with its corresponding ensembl-ID ENSG00000068024, PPP2R5A as ENSG00000066027 and MAP2K7 as ENSG00000076984. These genes are overexpressed in all three datasets of general glioma, glioblastoma multiforme as well as low-grade astrocytoma. Histone deacetylase 4 (HDAC4) is involved in deacetylation of histones, leading to a repression of transcription [62]. Protein phosphatase 2 regulatory subunit B56 alpha (PPP2R5A) has been implicated in a variety of regulatory processes, including cell growth and division, muscle contraction, and gene transcription based on the regulatory mechanism, protein phosphorylation, which is commonly employed in multiple cellular processes such as cell cycle progression, growth factor signaling, and cell transformation [62]. Mitogen-activated protein kinase kinase 7 (MAP2K7) acts as an essential component of the MAP kinase signal transduction pathway [63]. The general function of mitogen-activated protein kinase (MAPK) cascades is to relay environmental signals to the transcriptional machinery in the nucleus and thus modulate gene expression [62].

**Figure 5:**
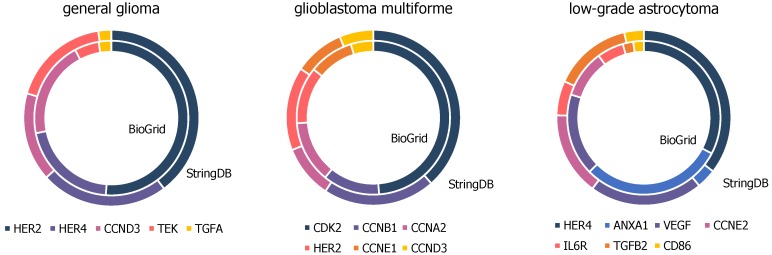
High-degree genes of general glioma, glioblastoma multiforme and low-grade astrocytoma samples, present in BioGrid and StringDB constructed networks upon top ten hubs.

There are several matching genes within the top hub nodes of networks constructed by BioGRID compared to the far smaller ones by StringDB, presented in Figure 5 and further details in Tables S2–S4. Top hub nodes from glioblastoma multiforme networks contain six matches, namely cyclin dependent kinase 2 (CDK2), cyclin A2 (CCNA2), cyclin B1 (CCNB1), erb-b2 receptor tyrosine kinase 2 (HER2), cyclin E1 (CCNE1), and cyclin D3 (CCND3). Five out of the ten top hub nodes from general glioma networks from StringDB and BioGRID are matching genes containing erb-b2 receptor tyrosine kinase 2 (HER2), erb-b2 receptor tyrosine kinase 4 (HER4), cyclin D3 (CCND3), TEK tyrosine kinase (TEK), and transforming growth factor alpha (TGFA). Seven out of the ten top hub nodes from both BioGRID and StringDB constructed networks of low-grade astrocytoma samples accord with each other, namely erb-b2 receptor tyrosine kinase 4 (HER4), annexin A1 (ANXA1), vascular endothelial growth factor A (VEGFA), cyclin E2 (CCNE2), interleukin 6 receptor (IL6R), transforming growth factor beta 2 (TGFB2), and CD86 molecule (CD86).

Some of the identified genes are hub nodes within clusters, as specified in Tables S2–S4. BioGrid constructed networks include several clusters. Top ten hub nodes of glioblastoma multiforme involve CDK2, HER2, CCNE1, HIF1A, CDKN1A and CDK1 as part of cluster 1. Further interrelation was given by CCNA2, CCNB1 and CCND3 belonging to clusters 3 and 4. In case of general glioma, top hub nodes included HER2 and HER4 as part of one cluster; as for astrocytoma, the top hub nodes listed CCNE2 and CDK2 as part of one cluster and VEGF and NRP1 as components of another cluster.

In case of the StringDB constructed networks, there were hardly any clusters isolated due to the smaller number of nodes and edges. Still, in case of glioblastoma multiforme, HER2 was identified to be a part of cluster 1 also involving other top hub nodes of proto-oncogenes KRAS, HRAS and NRAS.

The epidermal growth factor receptor (EGFR, HER1) is a major regulator of proliferation in tumor cells and a well-known target in glioblastoma treatment [64]. The human EGFR related family of receptor tyrosine kinases (HER) includes next to HER1/EGFR, additional members HER2 to HER4. The latter genes are well studied for their involvement in aberrant signaling of breast cancer but also within other tumors including glioma [52]. ANXA1 is also known to be involved in proliferation, differentiation and apoptosis, serves as a substrate for EGFR, and is implicated in tumor progression of astrocytoma [65]. VEGFA is involved in promoting tumor-induced angiogenesis and implicated with brain tumor progression [66]. Transforming growth factor receptor beta (TGFB) is likewise involved in the regulation of cell proliferation and postulated as a target for glioma therapy [67]. IL6R expression has been shown as a predictor of poor survival in glioma effecting tumor progression [68]. CD86 as ligand for the costimulatory receptor CD28 is involved in intratumoral T-cell stimulation [69,70]. TEK is known for glioma tumor progression [71]. Cyclins as key cell cycle factors are part of the regulation machinery behind tumor cell proliferation [72].

### 2.2. Comparison and Mapping of Different Database-Constructed Graphs

We compared StringDB to BioGRID networks by mapping BioGRID table columns via Biomart different node identifiers. While, by using Ensembl gene identifiers, we could find 41 similar nodes within both glioblastoma multiforme networks, by using the Ensembl protein identifiers, we could find 42 matching nodes, and by using BioGRID’s human readable label within Cytoscape to map with StringDB’s label provided by NetworkAnalyst, similar to HUGO Gene Nomenclature Committee (HGNC) symbols, we found 137 intersecting nodes between the two GBM networks.

We then tried to use Cytoscape’s build-in column mapping tool by mapping HGNC symbols to Ensembl Gene IDs. Since HGNC labels are written differently depending on database, format and tool, this mapping is far from comprehensive. We further tried to map Entrez gene ids to Ensembl and could map over 80% of the nodes. Missing 30 Ensembl gene identifier were added manually to the Ensembl gene id column of the imported network from Biogrid. The column was then complete but one gene. Moreover, ensembl has limitations, such as there being no ensembl gene id for the recombining binding protein suppressor of hairless pseudogene 3 (RBPJP3), though of no relevance in our use case. We experienced comparable column mapping results insofar as mapping HGNC symbols resulted only in less than half of the Ensembl gene identifier, while adding them up with Entrez mappings to complete nearly all (in glioma, only eight had to be added manually and in astrocytoma, also only eight).

We further merged disease-specific networks from StringDB and BioGrid, respectively, by using the Ensembl Gene Id as intersecting nodes. Table 2 shows the consensus results. In the case of GBM, the resulting intersection network counts 411 unconnected nodes, general glioma intersections resulted in 301 unconnected nodes, and astrocytoma intersections resulted in 285 unconnected nodes. The complete node lists as well as Cytoscape source files can be found at https://github.com/schokine/Cytoscape-glioma-CF.

Top hub nodes, for each glioma sub-type presented in Tables S2–S4, were compared manually in order to circumvent mapping difficulties between the various gene identifiers.

## 3. Discussion

The construction of PPI networks on the basis of overexpressed genes within disease samples can vary according to the applied tools and web resources. The extensive datasets on gene expression within chosen cancer samples posed the first challenge. These data had to be scaled down in order to be applicable as input for the diverse tools. Filtering data to a required minimum expression value will reduce entries. But it has to be kept in mind that low changes in expression levels can still be significant and could present a relevant or even crucial biological impact. Data entries in the five to six-digit range exceeded computing power and resources for online information retrieval from data repositories by the selected tools. In order to downsize sample data entries, we concentrated genes associated to the general biological function of proliferation as basic principle corresponding to cancer. This filtering step is one of many possibilities to analyse selected datasets in more detail. Other biological functions as apoptosis or migration could function likewise as approach to answer various questions for cancer research.

We constructed our PPI networks on expression profiles from upregulated genes within disease samples. Likewise, PPI graphs could be compared on the basis of downregulated genes which could equally possess significant biological impact such as regulation of apoptosis and prevent an imbalance of proliferation.

Differential graph analysis allowed us to compare the differences as well as similarities among datasets of selected disease types. GO analysis of dissected sample networks could highlight unique characteristics to disease subtypes and even point to possible biomarkers or novel molecular targets for treatment.

Calculating PPI networks from gene lists is based on estimations and therefore connected to an uncertainty [39,73]. We confined the PPI networks in NetworkAnalyst to only those interactions between proteins that are based on evidence at a high confidence level. In case of BioGRID, PPI networks data were refined to human taxonomy only while Cytoscape did not offer an option to choose between confidence scores or types of interaction evidence from BioGRID imported networks. However, all the respective StringDB and Biogrid-based networks share common subgraphs that show some similarities between the different database approaches, while the list of intersecting nodes could also be used for further analysis.

Hubs presenting high degree nodes can be used to evaluate signaling key points within samples. These can be of interest for understanding molecular mechanisms up to possible targets for treatment. Still, well-known and highly investigated proteins will be more likely assigned to a multiplicity of interactions than unstudied proteins. Thus, nodes with low number of interactions or even isolated outliers could hold relevant biological function within the disease. Results from corresponding BioGRID and StringDB high degree nodes list several genes that have been investigated to a certain extent or have been postulated to be involved in glioma signaling. The difference in top hubs between low-grade and high-grade glioma in comparison to general glioma can further point to tumor progression and malignancy. Further studies on unconnected nodes seem to be equally of interest. Differential graph analysis pinpoints three example genes which could be essential to glioma signaling. They have been indicated as possible prognostic biomarkers in different cancer diseases and require further evaluation respectively. Comparative Toxicogenomics Database lists HDAC4 an PP2R5A listing inferred gene-disease associations to glioma, mainly curated via chemicals, and no association between glioma and MAP2K7. Hub nodes within clusters could point to protein complexes since numerous proteins function within interacting aggregates. These interactions relate to biological functions and can be pointed out as signaling steps in disease. The analysis of the first ten top high-degree nodes already depicted several possible interacting complexes such as HER-signaling interrelated with cell cycle proteins which have been given much attention as targets in cancer treatment [74]. The examples of HER2 and CDK1/2 as well as CDKN1a and CCNE1 has yet to be evaluated. Expression of HER2 and HIF1A has been shown to correlate within other cancer subtypes [75]. Findings in general glioma samples of HER2 and HER4 to be part of the same protein cluster go along with studies on childhood medulloblastoma and other cancer subtypes showing coexpression of the two proteins and further receptor heterodimerization between them [76,77]. Cluster analysis within astrocytoma samples listed only VEGF and NRP1 within one complex. The corresponding proteins have been identified to interact in tumor biology and suggested as antitumor targets [78]. The only result from StringDB based graphs for possible protein complexes within glioblastoma multiforme listed HER2 and several RAS proto-oncogenes. HER2 gene amplification has been shown in glioblastoma multiforme in correlation to KRAS and NRAS mutations [79,80].

We further included data from a comparison of gene expression profiles of anaplastic glioma with or without the mutated IDH1/2 gene [81]. Using the above described protocol, these data included only three upregulated genes which are related to proliferation. These IDH wildtype and mutant specific data were not suitable for the comparison and analysis process described above, since the graph analysis resulted in small networks created by BioGrid and StringDB, which are shown in Figure S3. Unfortunately, we could not find additional gene expression data related to IDH mutation specifically. In this case, future experiments will be necessary to retrieve relevant results on network changes related to IDH-gene mutation using the above-described method.

Still, most of the example signaling components have been evaluated within various non-brain tumors and yet have to be studied in more detail for the various glioma diseases.

## 4. Methods

The main idea is to compare gene expression in general glioma including GBM and low-grade astrocytoma via graph-based analysis of different PPI databases using network analysis tools. At first, we use EMBL-EBI Expression Atlas [20] to download glioblastoma data from the PanCancer Analysis of Whole Genomes (PCAWG) [82] dataset. By filtering data from homo sapiens in the region of brain and by the selected disease types, we then took out the cutoff expression level < 3, and downloaded data in fragments per kilobase of transcript per million mapped reads (FPKM), a well-established normalized expression data format that can be found in many datasets provided as open data. This cutoff range is similar to a cutoff level < 10 for transcripts per million (TPM). Data were further filtered by GO biological function [83] in order to reduce entries within datasets. PantherDB [84] was used to get GO information on selected genes to filter on the term “proliferation”.

We used NetworkAnalyst [57] and Cytoscape [61] to create and visualize a PPI network for comparison for further analysis of selected genes. Cytoscape PPI construction and enrichment was processed via BioGRID using human taxonomy results only. NetworkAnalyst PPI construction and enrichment was based on StringDB at a confidence level of 90%.

We manually filtered the BioGRID-based networks by human nodes within Cytoscape by filtering on the column taxonomy id 9606 for human. We further processed the network data by using gene name mapping to PPI data via BIomart Martview service (available online: www.biomart.org/biomart/martview) and g:Profiler g:convert (available online: https://biit.cs.ut.ee/gprofiler/convert) including the removal of duplicates, and also used the column mapping functions in Cytoscape. In order to compare data for glioblastoma multiforme to other projects, we repeated the same steps, downloading the data for the disease types of general glioma data from the PCAWG study [82] and low-grade astrocytoma data from Expression Atlas [10,85], detailed in the beneath Section 4.2. Network clustering was computed via ClusterOne app within Cytoscape. BinGO app was used for gene ontology analysis of significant biological functions within created networks. Relevant gene results from network analysis were checked within the Comparative Toxicogenomics Database [86,87] for possible gene-disease associations.

### 4.1. Data Sources

Genomic data on glioblastoma multiforme, general glioma and low-grade astrocytoma and anaplastic glioma with or without the mutated IDH1/2 gene were obtained from EMBL-EBI Expression Atlas [20] from the PCAWG dataset [82], E-MTAB-3708 [85] and E-GEOD-52942 [81]. Glioma data were filtered on expression level cutoff 3 and FPKM data format in experiment E-MTAB-5200, on 30 September 2019 at 20:01:31. Glioblastoma data were filtered on expression level cutoff 3 FPKM in experiment E-MTAB-5200, on 22 September 2019 at 14:13:19. Astrocytoma data were filtered on expression level cutoff 3 FPKM in experiment E-MTAB-3708, on 30 September 2019 at 19:44:01. Gene expression profiles of anaplastic glioma with or without the mutated IDH1/2 gene were downloaded as a table of differentially expressed genes with *p*-value < 0.05 (DESeq) and log2fold cutoff 1 in experiment E-GEOD-52942. The list of genes was further filtered by upregulated genes only, on 20 Dec 2019 at 20:02:12.

### 4.2. Software and Web Resources

- AmiGO version 2.5.12 [88]
- BinGO version 3.0.3 [89]
- Biomart, Ensembl release 98, 2019-09 [90]
- ClusterOne version 1.0 [42]
- ClusterViz version 1.0.3 [91]
- Comparative Toxicogenomics Database, revision 15923 [86]
- Cytoscape version 3.7.2 [92]
- EMBL-EBI Expression Atlas release 31, 2019-05 [10]
- Gene Ontology release 2019-07-01 [84,93]
- g:Profiler release 2019-10-02 [94]
- NetworkAnalyst release 2019-10-07 [57]
- Omicsnet release 2019-08-07 [59]
- PantherDB version 14.1 [84]

Generated data can be found on https://github.com/schokine/Cytoscape-glioma-CF.

## 5. Conclusions

There are several open-access resources available for cancer research. We present and compare exemplary web-based and stand-alone tools for differential network analysis using open gene expression data on various glioma diseases as input. This approach highlights disturbed signaling components in general glioma, glioblastoma multiforme, as well as low-grade astrocytoma.

## Figures and Tables

**Table 1:** Differences between constructed networks from StringDB and BioGRID: counts of nodes and edges as well as cluster analysis.

Samples	Nodes	Edges	Clustering Coefficient	Connected Components	Network Diameter	Isolated Nodes
BioGRID-based networks computed by Cytoscape						
glioblastoma multiforme	2507	6108	0.076	4	9	3
general glioma	1907	4100	0.063	4	8	3
low-grade astrocytoma	1842	3813	0.035	4	8	3
merged intersection	1718	3439	0.008	14	9	13
merged union	2567	6340	0.076	4	9	3
StringDB-based networks computed by NetworkAnalyst						
glioblastoma multiforme	538	911	0.229	5	9	0
general glioma	392	559	0.130	6	10	0
low-grade astrocytoma	368	489	0.103	5	12	0
merged intersection	333	379	0.048	18	10	10
merged union	565	1005	0.217	3	12	0

**Table 2:** Merge Results from StringDB and BioGRID-based disease-specific networks.

Samples	Intersecting Nodes	Union of Nodes
glioblastoma multiforme	411	2634
general glioma	301	1997
low-grade astrocytoma	285	1949

## Supplementary Materials

Supplementary materials can be found at https://www.mdpi.com/1422-0067/21/2/547/s1.

## Abbreviations

ANXA1	annexin 1
CDK	cyclin-dependent kinase CCNA/B/D/E
EGFR	epidermal growth factor receptor
GBM	glioblastoma multiforme
GDC	Genomic Data Commons
GO	Gene Ontology
HDAC4	histone deacetylase
HER2/HER4	erb-b2 receptor tyrosine kinase 2/4 HGG
ICGC	International Cancer Genome Consortium
IDH1	isocitrate dehydrogenase 1
IL6R	interleukin 6 receptor
LGG	low grade glioma
MAP2K7	mitogen-activated protein kinase kinase 7 MAPK
NCI	National Cancer Institute
TCGA	The Cancer Genome Project
PPI	Protein Protein Interaction
PCAWG	Pancancer Analysis of Whole Genomes
PPP2R5A	protein phosphatase 2 regulatory subunit B56 alpha TEK
TGFA/TGFB	transforming growth factor alpha/beta TP53
TPM	transcripts per millions
FPKM	fragments per kilobase of transcript per million VEGFA
