# Supplementary material for: Open Data for Differential Network Analysis in Glioma
Source: Int J Mol Sci. 2020 Jan 15;21(2):547. doi: 10.3390/ijms21020547 (PMC7013918; doi:10.3390/ijms21020547)
Supplement: Supplementary file 1 [file ijms-21-00547-s001.pdf]

Supplementary

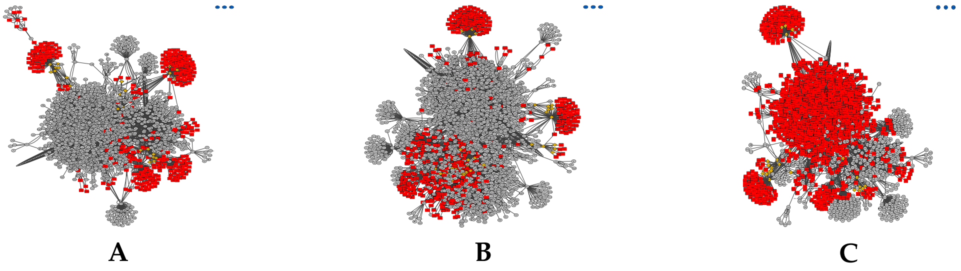

Figure S1. Graph clusters from BioGRID. enriched gene expression data on (**A**) general glioma, (**B**) glioblastoma multiforme, and (**C**) low-grade astrocytoma, filtered by gene ontology term “proliferation” related genes: Cytoscape visualization of enrichment using BioGRID database, rendered with Prefuse Force Directed Layout, clustered by ClusterOne (grey: outlier, yellow: overlap, red: cluster, blue: unassigned/default).

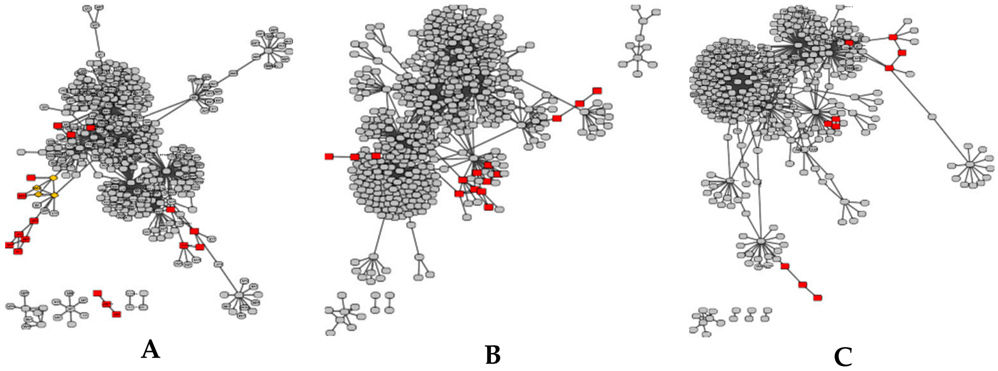

Figure S2. Graph clusters from StringDB. enriched gene expression data on (**A**) general glioma, (**B**) glioblastoma multiforme, and (**C**) low-grade astrocytoma, filtered by gene ontology term “proliferation” related genes: Cytoscape visualization of enrichment using NetworkAnalyst via StringDB, clustered by ClusterOne (grey: outlier, yellow: overlap, red: cluster).

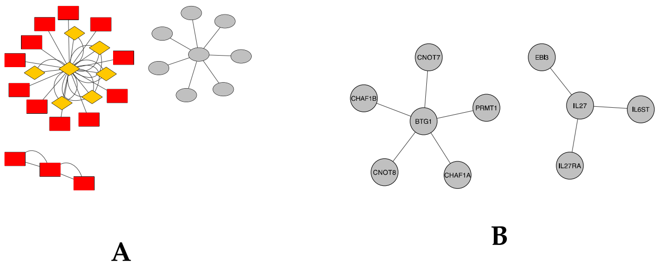

Figure S3. PPI graph on anaplastic glioma with or without the mutated IDH1/2. enriched gene expression data, filtered by gene ontology term “proliferation” related genes: Cytoscape visualization of enrichment using (**A**) via BioGRID database, (**B**) via NetworkAnalyst with StringDB, both clustered by ClusterOne (grey: outlier, yellow: overlap, red: cluster).

Table S1. Exemplary significant GO-terms within PPI networks of glioblastoma multiforme, general glioma and low-grade astrocytoma: based on elevated expression levels of genes associated with GO-term "proliferation"; significance expressed as p-value, calculated by BinGO.

Cancer Type	GO Biological Process	*p*-value
Glioblastoma multiforme	regulation of cell cycle	$1.05*10E^{-44}$
	post-translational protein modification	$5.82*10E^{-44}$
	regulation of phosphorylation	$1.03*10E^{-29}$
General glioma po	regulation of cell cycle	$3.49*10E^{-33}$
	st-translational protein modification	$5.03*10E^{-46}$
	regulation of phosphorylation	$4.35*10E^{-30}$
Low-grade astrocytoma	regulation of cell cycle	$1.37*10E^{-29}$
	post-translational protein modification	$3.82*10E^{-45}$
	regulation of phosphorylation	$1.68*10E^{-31}$

Table S2. High degree nodes within general glioma: comparison of top ten hubs in BioGRID and StringDB constructed networks, with degree as number of interactions, gene identifiers, component of cluster.

BioGrid			StringDB		
**Degree**	**Gene**	**Cluster No.**	**Degree**	**Node**	**Cluster No.**
289	ERBB2/HER2	1	66	ERBB2/HER2	1
117	ERBB4/HER4	1	39	ERBB4/HER4	na
115	CCND3	2	30	TEK	na
36	INCA1	10	27	CCND3	na
31	P8/NUPR1	7	12	TGFB3	na
29	TIE-2	na	4	TGFA	na
16	CDK4	na	3	KRAS	1
15	TGFA	na	3	HRAS	1
12	CDKN1A/CIP1	na	3	GRB2	na
11	CDK6	na	3	NRAS	1

Table S3. High degree nodes within glioblastoma multiforme: comparison of top ten hubs in BioGRID and StringDB constructed networks, with degree as number of interactions, gene identifiers, component of cluster.

BioGrid			StringDB		
**Degree**	**Gene**	**Cluster No.**	**Degree**	**Node**	**Cluster No.**
1178	p33/CDK2	1	165	CDK2	na
314	CCNA2/CCN1	4,3	89	CCNB1	na
290	CCNB1/CCNB	4,3	66	ERBB2/HER2	na
289	CD340/ERBB2/HER2	1	42	CCNA2	na
228	CCNE1	1	40	CCNE1	na
115	CCND3	4,3	38	CCNB2	na
107	LPC1/ANXA1	27,13	27	CCND3	na
100	CDKN1A/CIP1	1	23	VEGFA	na
66	HIF1A	1	16	TGFB2	na
83	P34CDC2/CDK1	1	12	TGFB3	na

Table S4. High degree nodes within astrocytoma: comparison of top ten hubs in BioGRID and StringDB constructed networks, with degree as number of interactions, gene identifiers, and component of cluster.

BioGrid			StringDB		
**Degree**	**Gene**	**Cluster No.**	**Degree**	**Node**	**Cluster No.**
117	ERBB4/HER4	2	39	ERBB4/HER4	na
107	LPC1/ANXA1	12	23	VEGFA/VEGF	na
62	MVCD1/VEGF	3	17	CCNE2	na
36	CYCE2/CCNE2	1	16	TGFB2	na
20	IL-6R-1/IL6R	11	7	IL6R	na
13	p33/CDK2	1	4	ANXA1	na
9	CCNI2	na	4	CD86	na
9	NRP1/NP1	3	3	STAT3	na
8	TGFB2	na	3	UBC	na
8	CD86	10	2	DCN	na
